# The probiotic yeast *Saccharomyces boulardii* CNCM I-745 prevents autophagy in macrophages and epithelial cells infected with *Vibrio parahaemolyticus*

**DOI:** 10.3389/fmicb.2026.1771497

**Published:** 2026-02-23

**Authors:** Rodolphe Pontier-Bres, Dorota Czerucka

**Affiliations:** Biomedical Department, Centre Scientifique de Monaco, Monaco, Monaco

**Keywords:** autophagy, infection, probiotic, *Saccharomyces boulardii* CNCMI-745, *Vibrio parahaemolyticus*

## Abstract

Although some pathogens neutralize autophagy to replicate within host cells, others exploit autophagy to induce cell death and lysis and thus acquire nutrients or escape the immune response. *Vibrio parahaemolyticus* (Vp, a marine bacterium that can infect humans) exerts cytotoxicity by inducing autophagy; this is followed by cell rounding and cell lysis. The probiotic yeast strain *Saccharomyces boulardii* CNCM I-745 (*S. boulardii*) has been shown to prevent infection by enteropathogenic bacteria. Here, we investigated the *in vitro* effects of *S. boulardii* on cytotoxicity and autophagy induced by Vp infection of two epithelial cell lines (HeLa and T84) and macrophage-like cells (RAW264.7). To that end, Vp-infected cells were exposed to *S. boulardii* cotreatment or pretreatment regimens. Pretreatment with *S. boulardii* of epithelial cells before infection by Vp was associated with a lower number of live intracellular bacteria, less Vp-induced morphological changes, and lower release of lactate dehydrogenase from infected cells. Importantly treatment with *S. boulardii* were associated with a lower degree of autophagy in Vp-infected epithelial cells and macrophage-like cells. Pretreatment and cotreatment with *S.boulardii* of epithelial cells downregulated the phosphorylation of the mitogen-activated kinases ERK1/2, JNK and p38 induced by Vp infection. In RAW264.7 cells, Vp infection induced the activation of JNK and p38 but only JNK phosphorylation was downregulated by *S. boulardii*. Taken as a whole, our data indicate that *S.boulardii* exerts an anticytotoxic effect on epithelial cells and prevents the Vp-induced autophagy of epithelial cells and macrophage-like cells. Our results suggest that the administration of probiotic *S.boulardii* CNCM I-745 could help to mitigate the effects of food-associated Vp infections.

## 1 Introduction

Probiotics are living microorganisms that, when administered in adequate amounts, have a beneficial effect on the host (Ouwehand et al., 2002; Sánchez et al., 2017). Most probiotics are bacteria; the best known are strains of *Lactobacillus* spp., and *Bifidobacterium* spp. (Saez-Lara et al., 2015). However, a non-bacterial microorganism has been classified as a probiotic agent: the yeast *Saccharomyces boulardii* CNCM I-745 (*S. boulardii*) (McFarland, 2010; Kazmierczak-Siedlecka et al., 2020). Several studies in animals and cell-based models have shown that *S. boulardii* has a beneficial effect by countering infections caused by various bacterial pathogens (including such as *Clostridioides difficile*, *Vibrio cholerae*, *Salmonella*, *Shigella*, *E. coli)*, viruses (rotavirus) and pathogenic yeasts [including *Candida albicans* (Kazmierczak-Siedlecka et al., 2020)]. In the context of a bacterial infection, *S. boulardii* acts either directly on bacterial virulence factors (e.g., toxins) or on the host’s intestinal mucosa and thus modulates the response to the pathogen (Czerucka and Rampal, 2019). *S. boulardii*’s protective action typically results from a combination of several complementary mechanisms. In *Salmonella* infections of intestinal epithelial T84 cells, for example, *S. boulardii* has been shown firstly to modify the intracellular signaling pathway implicated in cell invasion and inflammation (Martins et al., 2010; Pontier-Bres et al., 2014). A second mechanism depends on a direct binding of *Salmonella* to the *S. boulardii* cell wall and thus a reduction in bacterial motility (Pontier-Bres et al., 2014).

*Vibrio parahaemolyticus* (Vp) is a Gram-negative bacterium found mainly in marine and estuarine environments (Daniels et al., 2000). It is a common cause of acute gastroenteritis in people having consumed contaminated, undercooked seafood (Daniels et al., 2000). Vp is endemic to South-East Asia, where seafood consumption is frequent. This bacterium is becoming an increasing health concern because the increase in temperatures worldwide (and especially in ocean temperatures) favors the spread of a pandemic strain detected in the United States and in Europe (Baker-Austin et al., 2013; Martinez-Urtaza et al., 2010). Vp infects the human intestinal epithelium and causes diarrhea, abdominal cramps, nausea, and vomiting. Intestinal epithelial responses to Vp infections include damage to epithelial cells, changes in tight junction function and structure, and activation of proinflammatory responses resulting in phagocyte infiltration (Qadri et al., 2003; Takahashi et al., 2002; Lynch et al., 2005).

Vp is part of a small group of Gram-negative pathogens that use type III secretion systems (T3SS1 and T3SS2) to activate signaling and thus promote cell damage (for a review, see Zhang and Orth, 2013; Lynch et al., 2005). T3SS1 is required for cytotoxicity in a tissue culture model, whereas T3SS2 has been linked to enterotoxicity (Park et al., 2004). T3SS1-mediated cytotoxicity is caspase-independent and involves the induction of autophagy, cell rounding, and subsequent cell lysis (Burdette et al., 2008).

Autophagy is an evolutionarily conserved process that is essential for maintaining cell homeostasis [for review Randall-Demllo et al., 2013)]. Intestinal epithelial autophagy is essential for the host’s defense against invasion by and dissemination of pathogenic bacteria (Benjamin et al., 2013). After internalization, the microorganism-containing vacuoles (phagosomes) fuse with lysosomes to form phagolysosomes in which the bacteria are destroyed. However, pathogens have evolved various strategies for avoiding the formation of phagolysosomes; these include modification of the recruitment of the autophagic marker microtubule-associated protein light chain 3 (LC3) to pathogen-containing phagosomes and latter’s fusion with lysosomes (Jiao and Sun, 2019). Vp is known to induce autophagy by delivering a dedicated T3SS-1 effector (VopQ) into the host cells (Burette et al., 2009). By inducing the autophagy of epithelial cells, bacteria force the latter to work for them: Vp evades phagocytosis, destroys the epithelial cells, and uses the released nutrients for its own proliferation (de Souza Santos et al., 2014). Furthermore, the release of inflammatory content causes the recruitment of innate immune cells, such as macrophages. By inducing autophagy, Vp kills macrophages and thereby avoids an innate immune response.

Mitogen-activated protein kinase (MAPK) is one of a group of serine/threonine kinases that are activated in mammalian cells in response to extracellular stimuli. Three major MAPK pathways have been identified in mammalian cells: the ERK1/2 pathway is involved in cell proliferation and differentiation, whereas the p38 and JNK pathways are activated in response to stress (Lewis et al., 1998). Several *Vibrio* spp. manipulate MAPK signaling and thus impair the host’s response to infection (Kim et al., 2008; Kim et al., 2009; Harrison et al., 2008). In the case of Vp, VopQ has been shown to activate MAPK in human epithelial cell lines (Caco-2 cells and HeLa cells) (Matlawska-Wasowska et al., 2010; Shimohata et al., 2011). JNK and ERK activation was correlated with a maximum Vp-induced cytotoxic effect that resulted in cell death, apoptosis, and autophagy.

The objective of the present study was to investigate the protective effect of *S. boulardii* in the context of Vp infections of epithelial cells and macrophages. In earlier reports from our group, we studied T84 human colonic crypt-like cells and demonstrated the protective effects of *S. boulardii* with regard to bacterial adhesion, bacterial invasion, inflammatory responses, and apoptosis during infection by *Salmonella enterica* serovar Typhimurium, enterohemorrhagic *E. coli*, and enteropathogenic *E. coli* (Czerucka et al., 2000; Dahan et al., 2003; Dalmasso et al., 2006; Martins et al., 2010). To the best of our knowledge, T84 cells have not previously been used in studies of Vp infection. Here, we showed that (i) the T84 cell line is a suitable model for tracking host cell responses to Vp infection and (ii) *S. boulardii* prevents the cytotoxic induction of autophagy in T84 cells and RAW264.7 macrophage-like cells infected with Vp.

## 2 Materials and methods

### 2.1 Microorganisms

A clinical isolate of Vp (RIMD2210633 serotype O3: K6) was kindly provided by T. Honda (Osaka University, Osaka, Japan). Vp was cultured at 30 °C in a 5 mL tube containing 1.5% NaCl Luria-Bertani (LB) medium (standard condition).

*Saccharomyces boulardii* CNCM I-745 was obtained as a commercial formulation (Ultra-Levure®, Biocodex, Gentilly, France). Lyophilized *S. boulardii* was rehydrated and cultured overnight in Halvorson minimal medium containing 2% glucose, under aerobic conditions with shaking at 37 °C. The *S. boulardii* cells were washed twice in phosphate-buffered saline (PBS) and adjusted to 10^8^ CFU/mL in DMEM before use.

### 2.2 Cell lines

The T84 human colonic crypt-like cell line and the HeLa cervical cancer epithelial cell line were obtained from the European Collection of Authenticated Cell Cultures (Salisbury, UK). The T84 culture medium was a 1:1 mixture of Dulbecco’s Modified Eagle Medium (DMEM) and Ham’s-F12 medium, supplemented with 50 μg/mL penicillin, 50 μg/mL streptomycin (Gibco, France), and 5% fetal bovine serum (FBS, Hyclone, GE Healthcare, France). HeLa cells were maintained in DMEM medium with antibiotics and 5% fetal bovine serum (Hyclone, France).

The murine macrophage-like cell line RAW264.7 was obtained from the American Type Culture Collection (Manassas, VA, USA) and cultured in DMEM (Life Technologies, France) supplemented with 10% heat-inactivated FBS, 2 mM L-glutamine, and antibiotics (100 U/mL penicillin and 100 μg/mL streptomycin; Gibco, France). FBS was heat-inactivated at 56 °C for 30 min. Cells were maintained at 37 °C in a humidified atmosphere containing 5% CO_2_ and were subcultured every 2 or 3 days.

### 2.3 Infection procedure in the presence of *S. boulardii*

Cells were seeded into six-well tissue culture plates at 10^6^ cells per well and maintained for 2 to 3 days under the corresponding standard cell culture conditions. Prior to infection, the culture media were changed to media not supplemented with FBS or antibiotics. Bacteria were grown overnight in 1.5% NaCl-LB broth, pelleted by centrifugation, re-suspended in DMEM/F12 medium, and added to T84, HeLa and RAW264.7 cells at a multiplicity of infection (MOI) of 10. After predetermined infection times, the bacteria were eliminated by several washes with cold, sterile PBS and use for different biological assays described further on.

For the administration of *S. boulardii*, two treatment protocols were applied: (i) a cotreatment protocol in which cells were exposed concomitantly to Vp (at an MOI of 10) and *S. boulardii* (1 × 10^7^ cells/well) for various times (as indicated in the figure captions) and (ii) a pretreatment protocol in which cells were additionally treated overnight (for ~18 h) with *S. boulardii* (1 × 10^7^ cells/well) prior to exposure to Vp (at an MOI of 10). It should be noted that in the present study, the term “pretreatment” was considered to additionally include the cotreatment phase, i.e., the *S. boulardii* used for cell treatment before the Vp infection remained present during the Vp infection. For more details, see Supplementary Figures S1, S2.

### 2.4 Western blotting

At the indicated time points, infected cells were washed with PBS, scraped at 4 °C in lysis buffer, solubilized for 30 min at 4 °C, and then centrifuged at 14,000 g for 20 min at 4 °C, as described previously (Dahan et al., 2003). The protein concentration of the supernatant was determinate using a colorimetric, Lowry-type assay (DC Protein Assay, Bio-Rad, Marnes-la-Coquette, France). Equal amounts (50 μg) of whole cell lysates were subjected to 12% SDS-PAGE. The proteins were transferred onto a polyvinylidene fluoride membrane (PVDF, Hybond-P, Amersham, Orsay, France) and incubated overnight at 4 °C with anti-phospho-ERK1/2, anti-phospho-p38, anti-phospho-JNK (Cell Signaling Technology), or anti-ERK2, anti-p38, anti-JNK (Santa Cruz Biotechnology) and horseradish-peroxidase-conjugated anti-rabbit antibodies (New England Biolabs, Evry, France). Autophagy was monitored by incubation with an anti-LC3B antibody (#2775 from Cell Signaling Technology), and protein loading was normalized against beta actin (#4970 Cell Signaling Technology). The presence of antibodies was revealed with an enhanced chemiluminescence detection system (ECL, Amersham).

After imaging the Western blot with the Chemidoc system (Bio-Rad, Marnes-la-Coquette, France), the data were exported and analyzed using ImageLab software (also from Bio-Rad). For quantification, we subtracted the background noise intensity from the value of “Adj. Volume (Int)” (the band intensity, in arbitrary units). For normalization, we calculated the ratio between the band intensity in the experiment and that obtained in the control; the control value was therefore set to 1.

### 2.5 Measurement of LDH release

HeLa cells were infected with Vp for 3 h in the presence of *S. boulardii*, as described above. LDH release into the cell culture supernatant was measured with Cytotoxicity Detection Kit (Takara Bio, Saint-Germain-en-Laye, France). The results were expressed as the percentage of the LDH released after total lysis of the HeLa cells by 1% triton ×100.

### 2.6 Adhesion and invasion assays

The degree of bacterial adhesion to HeLa cells in the presence or absence of *S. boulardii* was quantified using the plate dilution method. Briefly, after 2 h of infection at an MOI of 10, the bacteria and *S. boulardii* present in the culture medium were eliminated by extensive washing with sterile PBS. Cells were lysed in water containing 0.1% bovine serum albumin. For the determination of bacterial invasion, washed monolayers were incubated for an additional hour with DMEM/F-12 containing 100 μg/mL of gentamicin and then another additional hour in DMEM/F-12 containing 10 μg/mL of gentamicin. Since gentamicin was not concentrated within the epithelial cells, intracellular bacteria survived the incubation, but adherent and extracellular bacteria were killed. The monolayers were then washed with sterile PBS, and epithelial cells with intracellular bacteria were lysed as described previously (Martins et al., 2010). The numbers of bacteria (adherent or intracellular) were evaluated by determination of the colony-forming units (CFU) after plating of different dilutions of the lysate onto agar medium. For more details, see Supplementary Figure S2.

### 2.7 Statistical analysis

**Figure 1:**
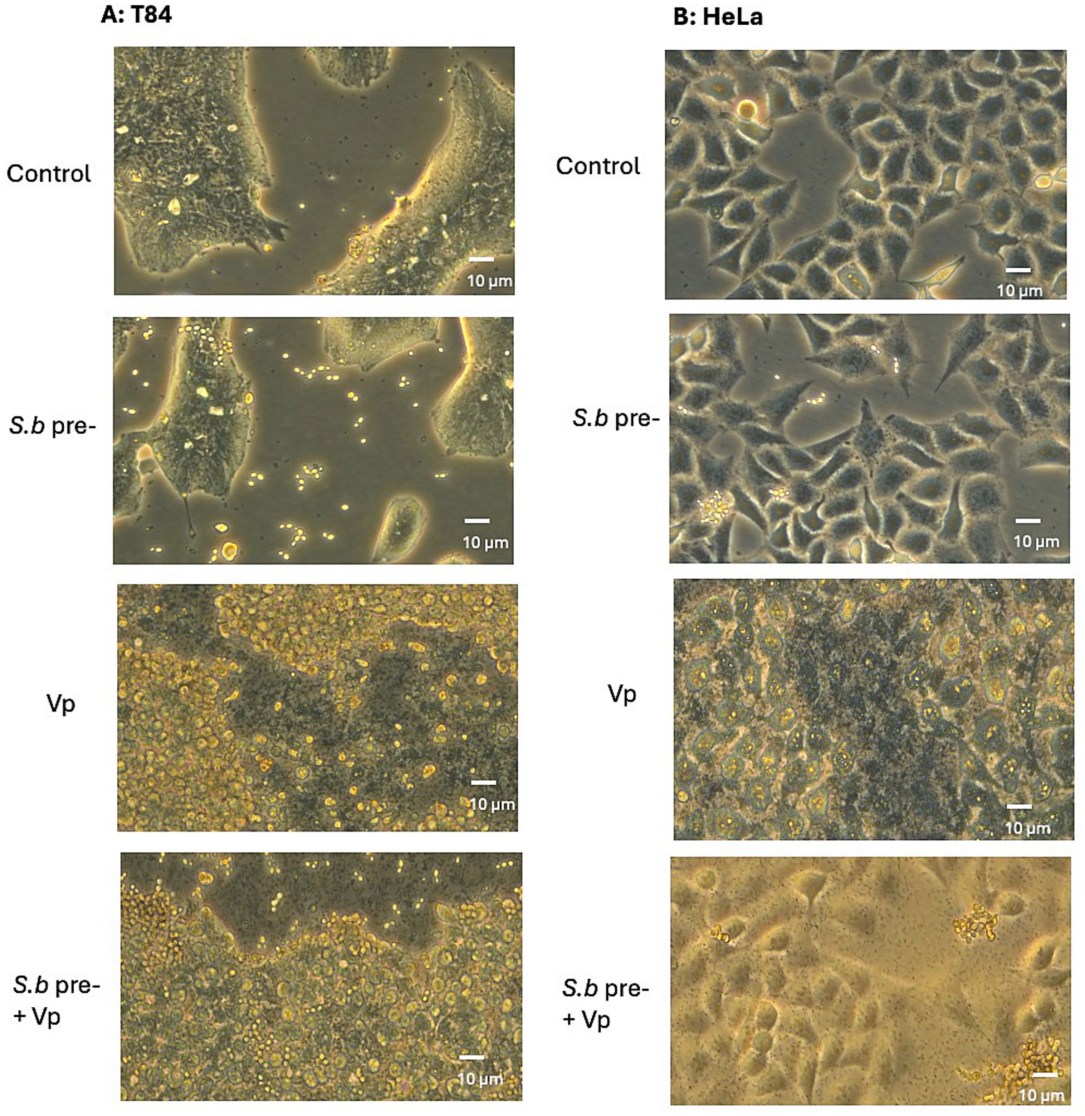
Pretreatment of *Vp*-infected T84 **(A)** and HeLa **(B)** cells with *S. boulardii* is associated with a lower level of cell rounding. Cells were incubated for ~18 h with *S. boulardii* alone, infected for 3 h with *Vp* alone, or pretreated with *S. boulardii* (i.e., before and during the *Vp* infection: “*Vp*+*S.b* pre-”). The control corresponds to non-infected cells. Cell monolayers were observed under a phase contrast light microscope.

Results are presented as the mean ± standard error of the mean (SEM). Data were analyzed using GraphPad Prism software (version 8, GraphPad Software LLC, Boston, MA, USA) using Student’s *t*-test with Bonferroni’s adjustment when comparing two groups (Table 1) or a one-way analysis of variance with Dunnett’s multiple comparison test when comparing more than two groups (Figure 1). The threshold for statistical significance was set to *p* < 0.05.

**Table 1:** Treatment of Vp-infected HeLa cells with *S. boulardii* is associated with a lower degree of cell invasion.

Cell treatment	Adhesion (×10^6^ CFU/well) Mean ± SEM	Intracellular bacteria (×10^4^ CFU/well) Mean ± SEM	% Invasion
Vp	99 ± 9.9	68 ± 12	0.68
*S.b* + Vp	98 ± 22	22 ± 2.9*	0 0.22*

HeLa cells were infected for 3 hours with Vp alone or pretreated with *S. boulardii* (i.e., before and during infection: “*S.b* pre-”). Cell invasion was assessed in a gentamicin protection assay. *Indicates a statistically significant difference (*p* < 0.05) versus Vp-infected cells alone (*n* = 3). CFU, colon y-forming units; SEM, standard error of the mean. Vp: *Vibrio parahaemolyticus*.

## 3 Results

### 3.1 Exposure to *S. boulardii* is associated with less rounding of Vp-infected epithelial cells

The cytotoxic effect of Vp infection on epithelial cells can be visualized by cell rounding. We assessed the morphological changes in T84 human epithelial cells and HeLa cells treated for different durations with Vp. After 3 h of infection, we observed the first morphological sign of cytotoxicity (cell rounding) in both cell lines (Figure 1). Pretreatment with. *S. boulardii* alone (i.e., in the absence of Vp) did not induce any morphological changes in either cell line. Hence, we found that pretreatment with *S. boulardii* lowered significantly cell rounding at 3 h post-infection (PI), indicating that the yeast protects cells from the cytotoxic effect of Vp.

### 3.2 Pretreatment of HeLa cells with *S. boulardii* is associated with a less intense cytotoxic effect of Vp infection

**Figure 2:**
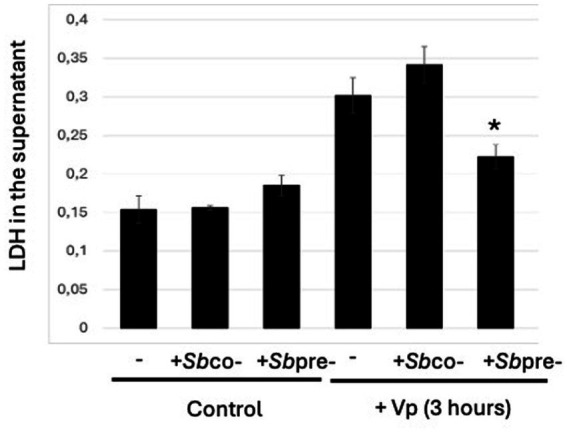
Pretreatment of HeLa cells with *S. boulardii* is associated with a less intense cytotoxic effect of Vp infection. The LDH release assay was performed in HeLa cells infected with Vp alone for 3 h, cotreated with *S. boulardii* (i.e., during the Vp infection “*S.b* co-”) or pretreated with *S. boulardii* (i.e., before and during the Vp infection: “*S.b* pre-”). Control cells were treated with *S. boulardii* for 3 h (*S.b* co-) or ~18 h (*S.b* pre-). *Indicates a statistically significant difference (*p* < 0.05) versus Vp-infected cells alone (*n* = 3).

To investigate the cytotoxic effect of Vp infection in HeLa cells, we used a lactate dehydrogenase (LDH) release assay to determine whether cell contents were released during infection. Firstly, we checked that exposure of cells to *S. boulardii* alone did not influence the level of LDH found in the extracellular medium, when compared with control cells (Figure 2). In contrast, we observed a higher level of LDH release into the extracellular medium after 3 h of HeLa cell infection by Vp. When cells were challenged with *S. boulardii* and Vp concomitantly for 3 h (as in the cotreatment protocol), the extracellular LDH level was similar to that observed for Vp-infected cells. Hence, a lower level of LDH release required prior incubation with *S. boulardii* (as in the pretreatment protocol) before Vp infection.

### 3.3 Exposure of HeLa cells to *S. boulardii* is associated with a lower degree of intracellular Vp survival

To determine whether *S. boulardii* modulates the adhesion of Vp and/or the bacterium’s intracellular survival, HeLa cells were treated with *S. boulardii* prior to and during infection by Vp (i.e., in the pretreatment protocol). Vp adhesion to HeLa cells was not significantly affected by *S. boulardii* treatment because the CFU count 2 h PI was similar to that observed for Vp-infected cells alone (*p* > 0.05). Hence, *S. boulardii* did not appear to interfere with bacterial adhesion (Table 1).

In contrast, the proportion of live intracellular bacteria 4 h PI (as assessed in the gentamycin protection assay) was significantly lower in *S. boulardii* treated groups (0.22%) than in Vp-infected cells alone (0.68%).

### 3.4 Treatment of Vp-infected T84 epithelial cells with *S. boulardii* is associated with a less intense autophagy response

**Figure 3:**
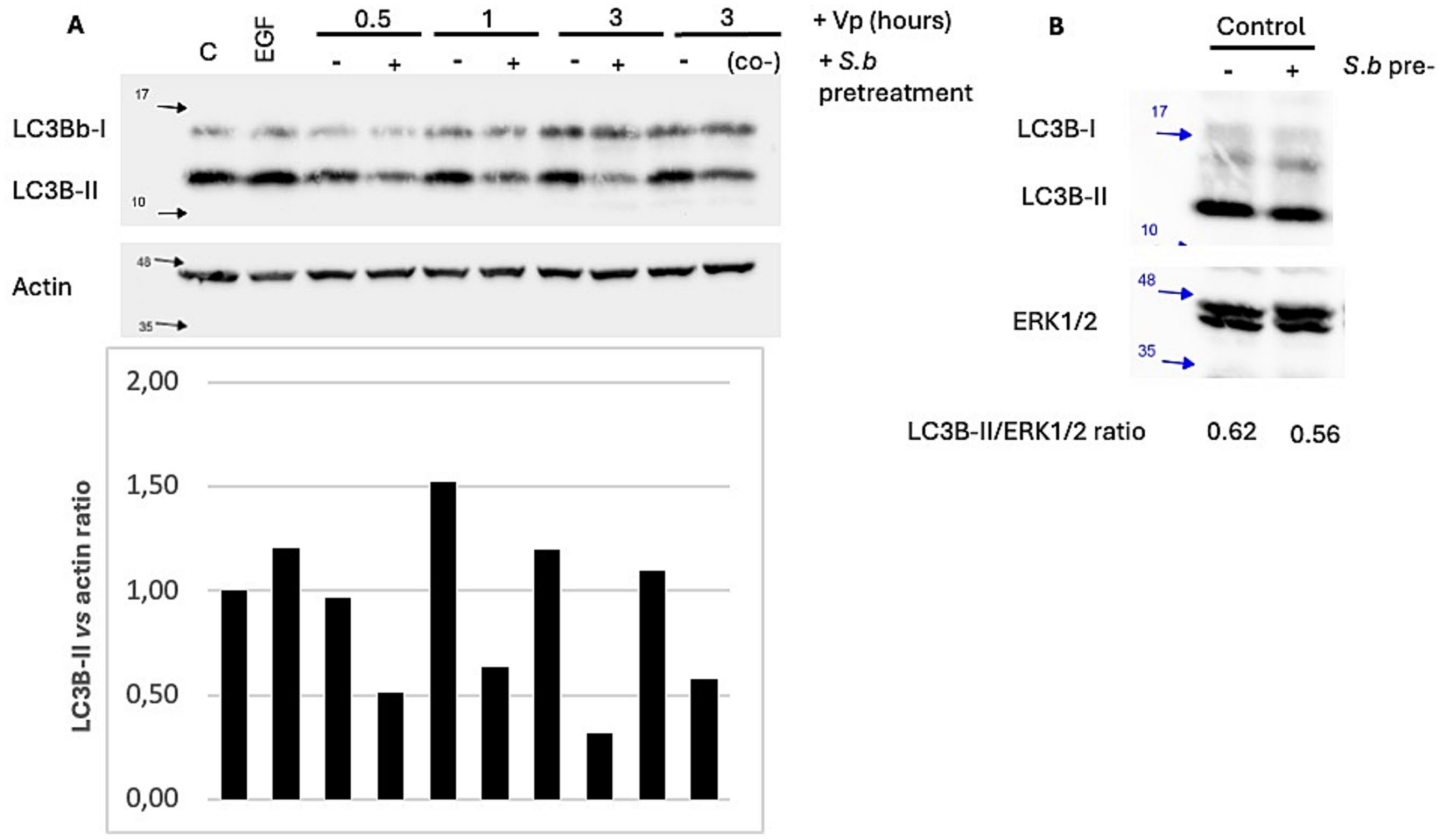
Treatment of T84 cells with *S. boulardii* is associated with a lower degree of Vp-induced autophagy. **(A)** T84 cells were infected for various durations with Vp alone, cotreated with *S. boulardii* (i.e., during infection only: “*S.b* co-”), or pretreated with *S. boulardii* (i.e., before and during infection: “*S.b* pre-”), “C” corresponds to control (non-infected) cells. **(B)** *S. boulardii* incubation for ~18 h did not influence autophagy of T84 cells in control, non-infected cells. Cell lysates were prepared and separated by SDS-PAGE. Proteins were transferred to a PVDF membrane, which was probed with anti-LC3B antibodies. The stripped membranes were also probed with actin or ERK antibodies, to control protein loading. A representative image of an LC3 immunoblot from T84 cells is shown (*n* = 4).

LC3 is an autophagy marker that resides in the cytosol (as LC3-I) under normal conditions. During recruitment to the autophagosome, LC3-I is lipidated to yield LC3-II, which binds to the autophagosome membrane (Kabeya et al., 2000; Jiao and Sun, 2019). We studied the effect of *S. boulardii* pretreatment and cotreatment on autophagy induction in epithelial cells exposed for various durations (30 min, 1 h, and 3 h) to Vp. The extracted proteins were immunoblotted with antibodies against LC3B-I and LC3B-II. To control the loading quantities, the stripped membranes were re-probed with anti-actin antibody. A Western blot analysis showed that Vp infection induced the accumulation of LC3B-II within 1 h and the level of LC3B-II remained high throughout the course of the Vp infection in T84 cells (Figure 3A) and HeLa cells (Supplementary Figure S3). In both epithelial cell lines, however, the autophagic response to the infection was modified by *S. boulardii*. After pretreatment with *S. boulardii*, autophagy was significantly less intense in T84 cells (Figure 3A) and was even completely abolished in HeLa cells (Supplementary Figure S3). Lastly, we checked that incubation of epithelial cells with *S. boulardii* alone did not induce autophagy (Figure 3B).

### 3.5 Treatment of Vp-infected RAW264.7 cells with *S. boulardii* is associated with a less intense autophagy response

**Figure 4:**
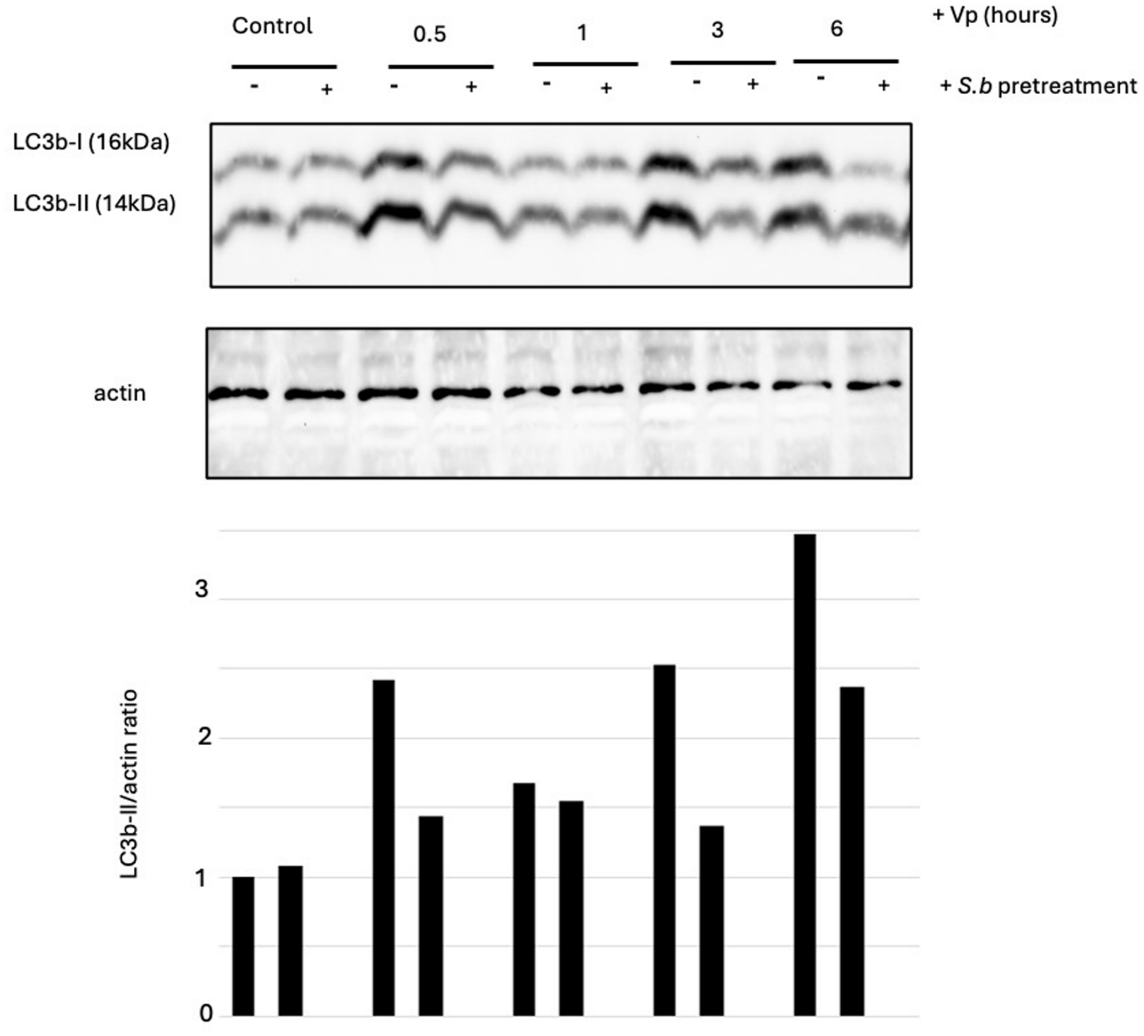
Treatment of RAW264.7 cells with *S. boulardii* is associated with a lower degree of Vp-induced autophagy. RAW264.7 cells were infected for various durations with Vp alone, or pretreated with *S. boulardii* (i.e., before and during infection: “*S.b* pre-”). The control corresponds to non-infected cells exposed (or not) to *S. boulardii* in the pretreatment condition. Cell lysates were prepared and separated by SDS-PAGE. Proteins were transferred to a PVDF membrane, which was probed with anti-LC3B antibodies. The stripped membranes were also probed with actin or ERK antibodies, to control for protein loading. A representative image of an LC3 immunoblot from RAW264.7 cells is shown (*n* = 3).

We studied the effect of *S. boulardii* pretreatment and cotreatment on autophagy induction in RAW264.7 macrophage-like cells exposed for various durations (30 min, 1 h, 3 h, and 6 h) to Vp. The extracted proteins were processed as described above. A Western blot analysis revealed that the conversion of LC3B-I to LC3B-II peaked at 6 h (Figure 4). As was the case for the T84 epithelial cells, pretreatment and with *S. boulardii* were associated with a significantly lower level of autophagy in infected cells at 6 h PI.

### 3.6 Treatment of epithelial cells with *S. boulardii* is associated with less Vp-induced phosphorylation of MAPK

**Figure 5:**
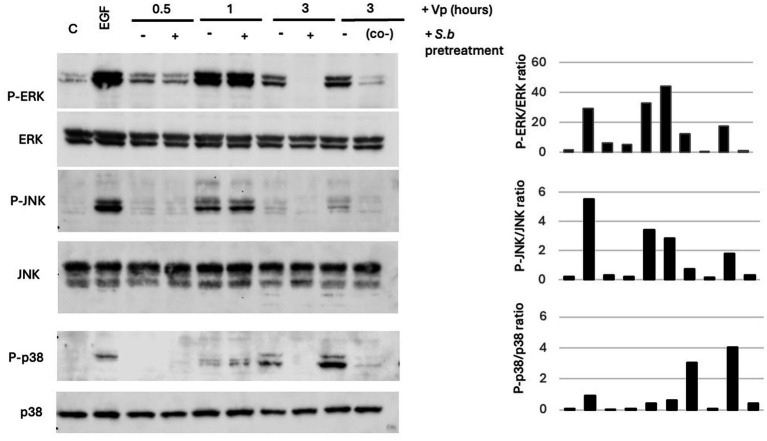
Treatment of *Vp*-infected T84 cells with *S. boulardii* is associated with a lower degree of MAPK activation. Cells were infected for various durations with *Vp* alone, cotreated with *S. boulardii* (i.e., during infection only: “*S.b* co-”), or pretreated with *S. boulardii* (i.e., before and during infection: “*S.b* pre-”). MAPK activation was measured at 0.5, 1, and 3 h PI. Epidermal growth factor (EGF) was used as a positive control for the induction of phosphorylation of each MAPK. “C” corresponds to control (non-infected) cells. Cell lysates were prepared and separated by SDS-PAGE. Proteins were transferred to a PVDF membrane, which was probed with antibodies against phospho-ERK, phospho-JNK, and phospho-p38. The stripped membranes were also probed with antibodies against total MAPKs, to control for protein loading. The image of MAPK immunoblots from T84 cells is representative of *n* = 3 experiments.

MAPK is activated by many pathogens when they colonize the host and induce inflammatory response and cytotoxicity (Krachler et al., 2011). In the context of infection of intestinal epithelial cells by Vp, activation of the MAPK has been investigated in the Caco-2 human cell line (Matlawska-Wasowska et al., 2010; Shimohata et al., 2011). In the present study, we used T84 cells to investigate the effect of Vp infection on MAPK. Cells were exposed for various durations to Vp: 30 min, 1 h, and 3 h. The extracted proteins were immunoblotted with antibodies against phospho-JNK, phospho-p38, and phospho-ERK1/2. To control the loading and measure total amounts, the stripped membranes were re-probed with antibodies against total JNK, p38 and ERK1/2. In T84 cells, we observed a transient activation of ERK1/2 and JNK with a peak at 1 h PI and a decrease by 3 h PI (Figure 5). For p38, activation of p38 occurred later (at 3 h PI). However, at 3 h PI, pretreatment with *S. boulardii* had shift down the activation of all three MAPKs. We also investigated MAPK activation in HeLa cells following Vp infection. ERK1/2 and p38 were activated as soon as 0.5 h PI (Supplementary Figure S4). Surprisingly, the levels of ERK1/2 and p38 phosphorylation were higher in HeLa cells pretreated with *S. boulardii* than in Vp-infected cells alone.

### 3.7 Treatment of RAW264.7 cells with *S. boulardii* is associated with less Vp-induced phosphorylation of JNK

**Figure 6:**
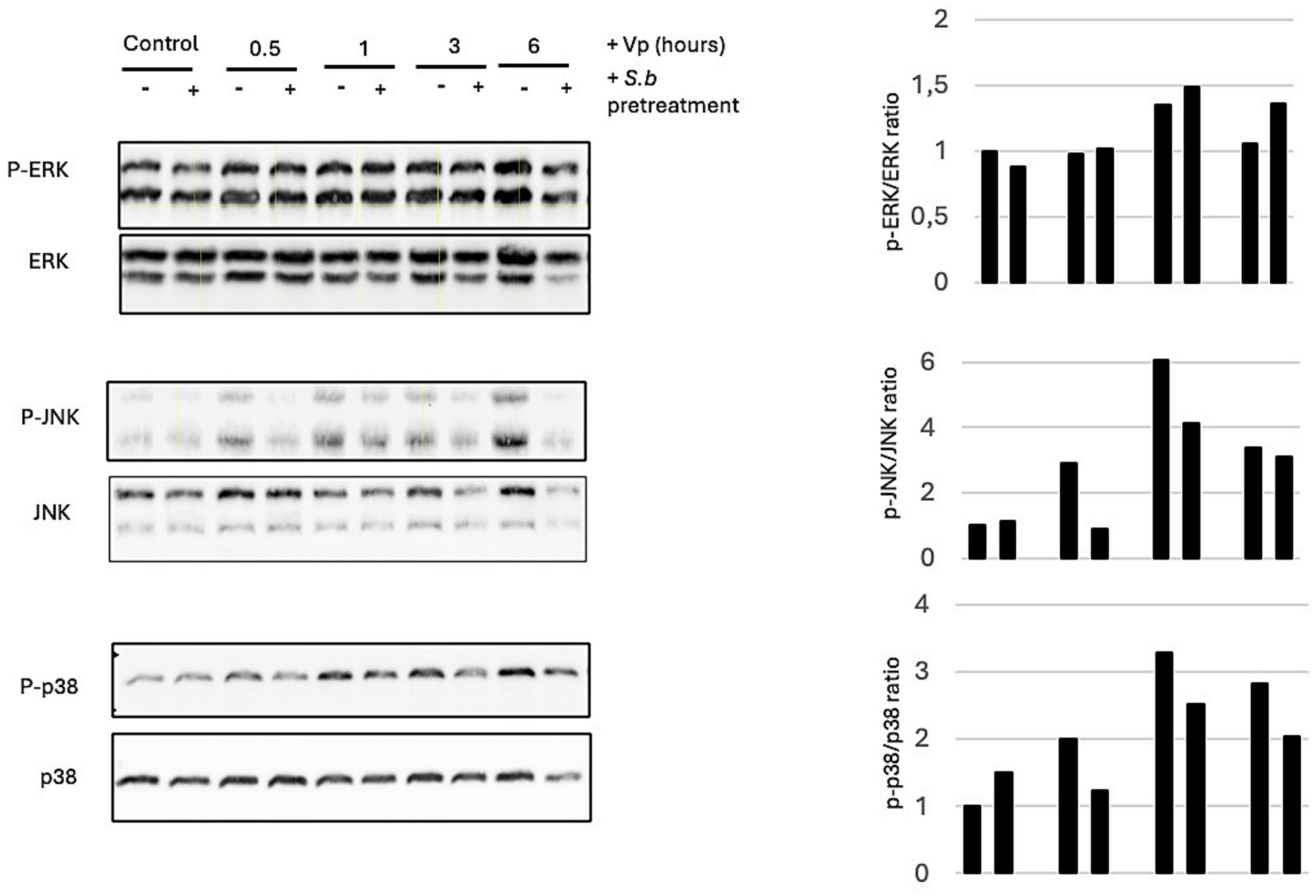
Treatment of Vp-infected RAW264.7 cells with *S. boulardii* is associated decreased JNK activation. Cells were infected for various durations with Vp alone or pretreated with *S. boulardii* (i.e., before and during infection: “*S.b* pre-”). The control corresponds to non-infected cells exposed or not to *S.b* (pretreated condition). JNK activation was measured at 0.5, 1, and 3 h PI. Cell lysates were prepared and separated by SDS-PAGE. Proteins were transferred to a PVDF membrane, which was probed with antibodies against phospho-ERK, phospho-JNK, and phospho-p38. The stripped membranes were also probed with antibodies against total MAPKs, to control for protein loading. The image of MAPK immunoblots from T84 cells is representative of *n* = 3 experiments.

To assess MAPK phosphorylation, RAW264.7 cells were exposed for different durations (30 min, 1 h, 3 h and 6 h) to Vp. The extracted proteins were processed as described above. Infection of RAW264.7 cells was not associated with the induction of ERK1/2 phosphorylation (Figure 6). Activation of JNK and p38 occurred as early as 30 min PI and increased until 6 h PI. At the time point of 6 h PI, only the activation of JNK had been shut down in cells pre-treated with *S. boulardii* before Vp infection. *S. boulardii* treatment was not associated with less phosphorylation of p38.

## 4 Discussion

Although Vp was long thought to be an extracellular pathogen, it is now known that the bacterium can invade non-phagocytic cells (Zhang et al., 2012). This activity was confirmed by our present data with HeLa cells; after the adhesion of Vp, a small proportion of the bacteria entered the cells. Treatment with *S. boulardii* did not influence the number of adherent bacteria but was associated with a smaller number of intracellular bacteria. This finding supports the hypothesis whereby *S. boulardii* can modify some of the intracellular signaling pathways involved in the survival of bacteria inside cells. Observations of the shape of T84 cells and HeLa cells showed that at 3 h PI, Vp infection induced cell rounding. However, in cells exposed to *S. boulardii* before Vp infection, the numbers of rounded T84 and HeLa were lower. These findings strongly suggest that *S. boulardii* prevents the cytotoxicity induced by Vp infection. This hypothesis was confirmed by the lower level of LDH release observed in cells infected with Vp in the presence of *S. boulardii*, relative to cells infected in the absence of *S. boulardii*.

In eucaryotic cells, autophagy is an important biological process for responding to nutrient stress and eliminating pathogenic bacteria. To survive, bacterial effectors use various strategies to interfere with host signaling pathways and avoid autophagosome-lysosome fusion (Jiao and Sun, 2019). Vp is one of the bacterial pathogens that hijacks the host cell’s autophagy pathway for its own benefit. The bacterium injects the T3SS-1 effector VopQ to induce autophagy in epithelial cells and macrophages (Burette et al., 2009). As shown in the present study, infection of HeLa cells by Vp was associated with greater conversion of LC3B-I to LC3B-II; this started as soon as 30 min after exposure of cells to Vp and continued at 1 and 3 h PI. Vp infection also induced an autophagic response in T84 cells, with greater conversion of LC3B-II observed at 1 h PI. Pretreatment and cotreatment of HeLa and T84 cells with *S. boulardii* abolished Vp-induced autophagy. This downregulation was observed in T84 cells co-treated with *S. boulardii* and Vp for just 3 h, suggesting that the yeast exerts its action rapidly. Taken as a whole, these data show that the administration of *S. boulardii* before or during infection can antagonize Vp-induced autophagy in epithelial cells. As reported in the literature, Vp uses autophagy to evade phagocytic vacuoles, survive in epithelial cells, and then destroy the cells for its own benefit (Burdette et al., 2008; Burette et al., 2009). Inhibition of Vp-induced autophagy by *S. boulardii* might therefore account for the lower count of intracellular bacteria and the inhibition of Vp-induced cytotoxic effects in both epithelial cell lines.

Induction of autophagy in RAW264.7 macrophage-like cells by Vp has been reported previously (Burdette et al., 2008). Our time-course experiments with Vp-infected RAW264.7 cells showed that the conversion of LC3B-I to LC3B-II peaked at 6 h PI. Pretreatment with *S. boulardii* were associated with a lower level of Vp-induced autophagy in the RAW264.7 cells at 6 h PI.

Taken as a whole, these data indicate that *S. boulardii* can inhibit Vp-induced autophagy in both epithelial and immune cells. In the context of bacterial infection, the protective effect of *S. boulardii* on the intestinal mucosa has been demonstrated in an *in vivo* model (Czerucka and Rampal, 2019).

Vp-induced cytotoxic and inflammatory responses are associated with the activation of the MAPK pathway in Caco-2 and HeLa human epithelial cell lines (Matlawska-Wasowska et al., 2010; Shimohata et al., 2011). Activation of JNK and ERK (but not p38) is involved in Vp’s ability to kill Caco-2 cells (Matlawska-Wasowska et al., 2010). By using the T84 model of intestinal epithelial cells, we found that Vp infection induced the phosphorylation of JNK, ERK and p38 with different time courses: ERK1/2 and JNK were phosphorylated at 1-h PI and p38 was phosphorylated at 3 h PI. The time course of MAPK phosphorylation in T84 cells was similar to that described in Caco-2 cell, although the levels of phosphorylation differed (Matlawska-Wasowska et al., 2010).

Pretreatment of T84 cells with *S. boulardii* before Vp infection inhibited the phosphorylation of the 3 MAPKs. These data agree with our previously published results, which demonstrated that *S. boulardii* can decrease the MAPK activation in T84 cells induced by pathogenic bacteria (Czerucka et al., 2000; Dahan et al., 2003; Dalmasso et al., 2006; Martins et al., 2010). As was the case for Vp-induced autophagy, Vp-induced phosphorylation of MAPKs was abolished in T84 cells co-exposed to *S. boulardii* and Vp for 3 h.

In HeLa cells infected with Vp, we observed activation of ERK1/2 and p38 as early as 30 min PI and then for 1 and 3 h PI. Curiously, the incubation of HeLa cells with *S. boulardii* before Vp infection did not induce the downregulation of MAPK activation. These data suggest that *S. boulardii*’s effect on MAPK phosphorylation is cell-type-dependent. One possible explanation for this difference relates to morphological aspects. As seen in Figure 2, HeLa cells grow as isolated cells and are more exposed to Vp. In contrast, T84 cells form clusters of cells and then multimonolayers and so are more difficult to access by Vp. Another possible explanation for this difference relates to the Vp T3SS1 effectors, which are essential for MAPK activation in epithelial cells. Reports by other researchers have shown that VopQ (VP1680) can differentially activate MAPKs in HeLa and Caco-2 cells (Matlawska-Wasowska et al., 2010). VopQ induced activation of JNK and ERK, which appeared to be important for the cytotoxic effect of Vp on Caco-2 cells. In HeLa cells, VopQ had a less important role; hence, the various cell lines might differ in their sensitivity to T3SS effectors. Moreover, as reported in the literature, the activation of MAPK in HeLa and Caco-2 cells is not abolished when VP1680 is non-functional, and so other T3SS1 effectors may activate this signaling pathway (Matlawska-Wasowska et al., 2010). Even in the presence of T3SS1 effectors, MAPK can be also activated by lipopolysaccharide (LPS) binding to Toll-like receptor 4. We investigate this possibility by incubating T84 cells with LPS and *S. boulardii*. The yeast had no effect on LPS-induced MAPK activation (data not shown). *Vibrio* spp. have been shown to produce flagellin - another candidate for MAPK activation via its binding to Toll-like receptor 5 (Harrison et al., 2008). In future research, it will be interesting to investigate *S. boulardii*’s effect on flagellin.

The differences in the data obtained in HeLa cells versus T84 cells suggests that *S. boulardii*’s effect depends not only on the cell type but also on the yeast’s action on Vp effectors involved in the activation of these MAPKs. Modification of virulence factors by *S. boulardii* has been reported in the context of *Citrobacter-rodentium*-induced colitis (Wu et al., 2008). The ameliorating effect of *S. boulardii* was associated with the less bacterial adhesion to the mucosa and less secretion of T3SS1 involved in adhesion through the intimin receptor Tir and the translocation apparatus EspB. We cannot rule out modification of Vp’s T3SS1 or T3SS2 effectors by *S. boulardii*.

## 5 Conclusion

Taken as a whole, the results of the present *in vitro* study show that *S. boulardii* can suppress the autophagy induced by Vp infection of epithelial cells and macrophage cells. *In vivo*, the probiotic yeast might therefore help to prevent the intestinal epithelium damage caused by Vp infection.

## Data Availability

The raw data supporting the conclusions of this article will be made available by the authors, without undue reservation.

## Supplementary material

SUPPLEMENTARY FIGURE S1 The pretreatment and cotreatment protocols for Western blot analyses of intracellular signaling. The figure shows a schematic representation of the treatment timelines applied to HeLa, T84 and RAW264.7 cells for immunoblot analysis. In the pretreatment protocol, the cells were exposed to the probiotic *S. boulardii* (*Sb*) for 18 hours prior to Vp infection and then during the infection. In the cotreatment protocol, the cells (HeLa and T84) were exposed to *S. boulardii* during the Vp infection only. After different times post infection (as indicated in the Figure legend) the cells were washed and protein was extracted for further immunoblot analysis.

SUPPLEMENTARY FIGURE S2 The pretreatment and cotreatment protocols for adhesion/invasion experiments. The figure shows a schematic representation of the treatment timelines applied to HeLa cells during adhesion/invasion experiments. In the pretreatment protocol, the HeLa cells were exposed to the probiotic *S. boulardii* (*Sb*) for 18 hours prior to Vp infection and then during the infection. In the cotreatment protocol, the cells were exposed to *S. boulardii* during the Vp infection only. After 2 hours of infection, gentamycin 100 μg/ml was added to cell medium for 1 hour, and then the cells were washed and exposed to fresh medium containing gentamycin 10 μg/ml for another hour.

SUPPLEMENTARY FIGURE S3 *Saccharomyces boulardii* treatment prevents autophagy in HeLa cells. of LC3b-II was quantified in HeLa cells infected with Vp alone or pretreated with *S. boulardii* (i.e. before and during infection: “*S.b* pre-”). The results are representative of at least three independent experiments.

SUPPLEMENTARY FIGURE S4 Activation of ERK1/2 and p38 in HeLa cells infected with Vp alone or pretreated with *S. boulardii*. Cells were infected for various durations with Vp alone or pretreated with *S. boulardii* (i.e. before and during infection: “*S.b* pre-”). ERK1/2 and p38 activation was measured at 0.5, 1 and 3 hours PI. Cell lysates were prepared and separated by SDS-PAGE. Proteins were transferred to a PVDF membrane, which was probed with antibodies against phospho-ERK and phospho-p38. The stripped membranes were also probed with antibodies against total MAPKs, to control for protein loading.
