# Supplementary figures and images for: The probiotic yeast Saccharomyces boulardii CNCM I-745 prevents autophagy in macrophages and epithelial cells infected with Vibrio parahaemolyticus
Source: Front Microbiol. 2026 Feb 23;17:1771497. doi: 10.3389/fmicb.2026.1771497 (PMC12968228; doi:10.3389/fmicb.2026.1771497)

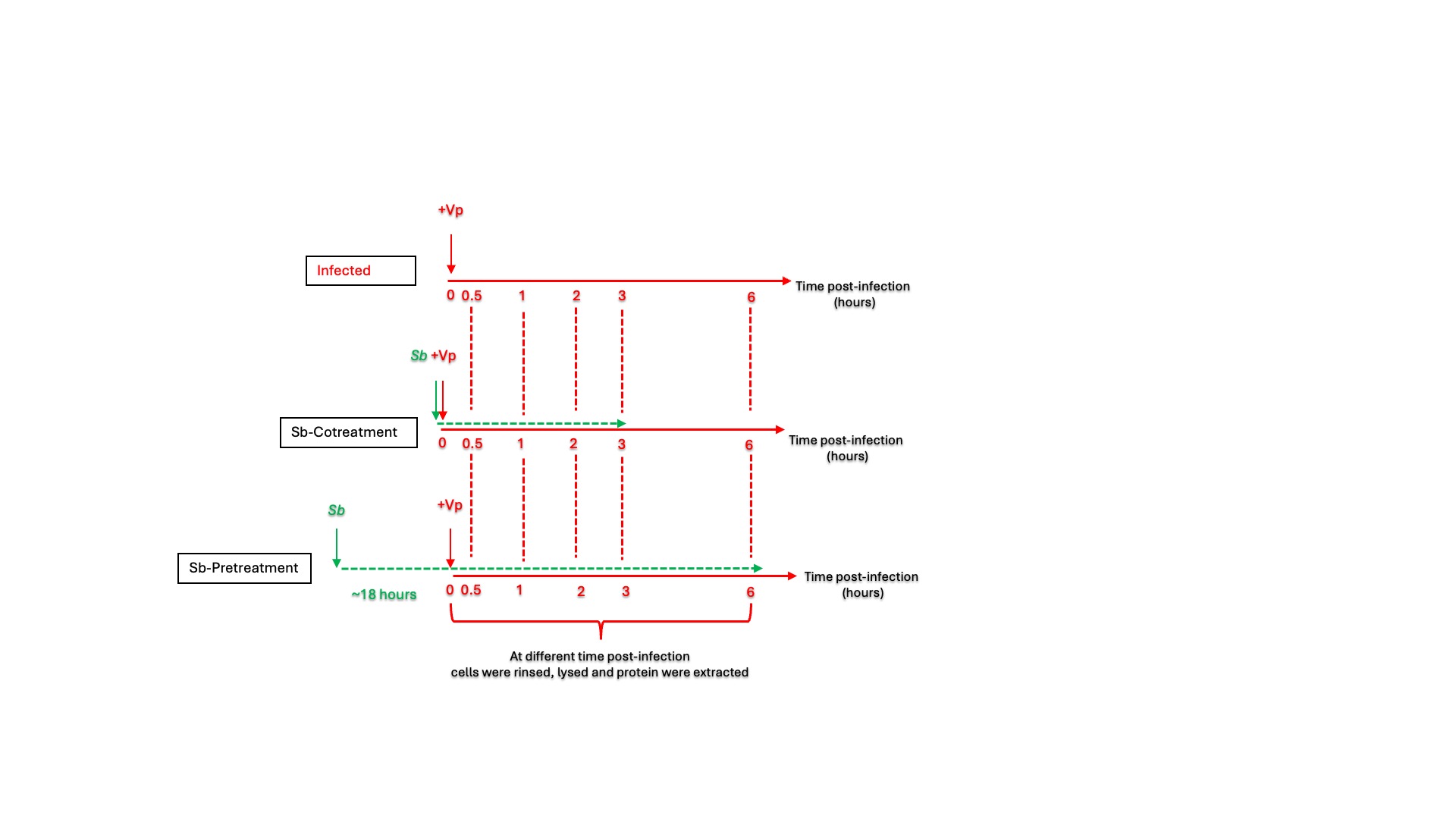

Supplement: Supplementary file 1 [file Image_1.jpeg]

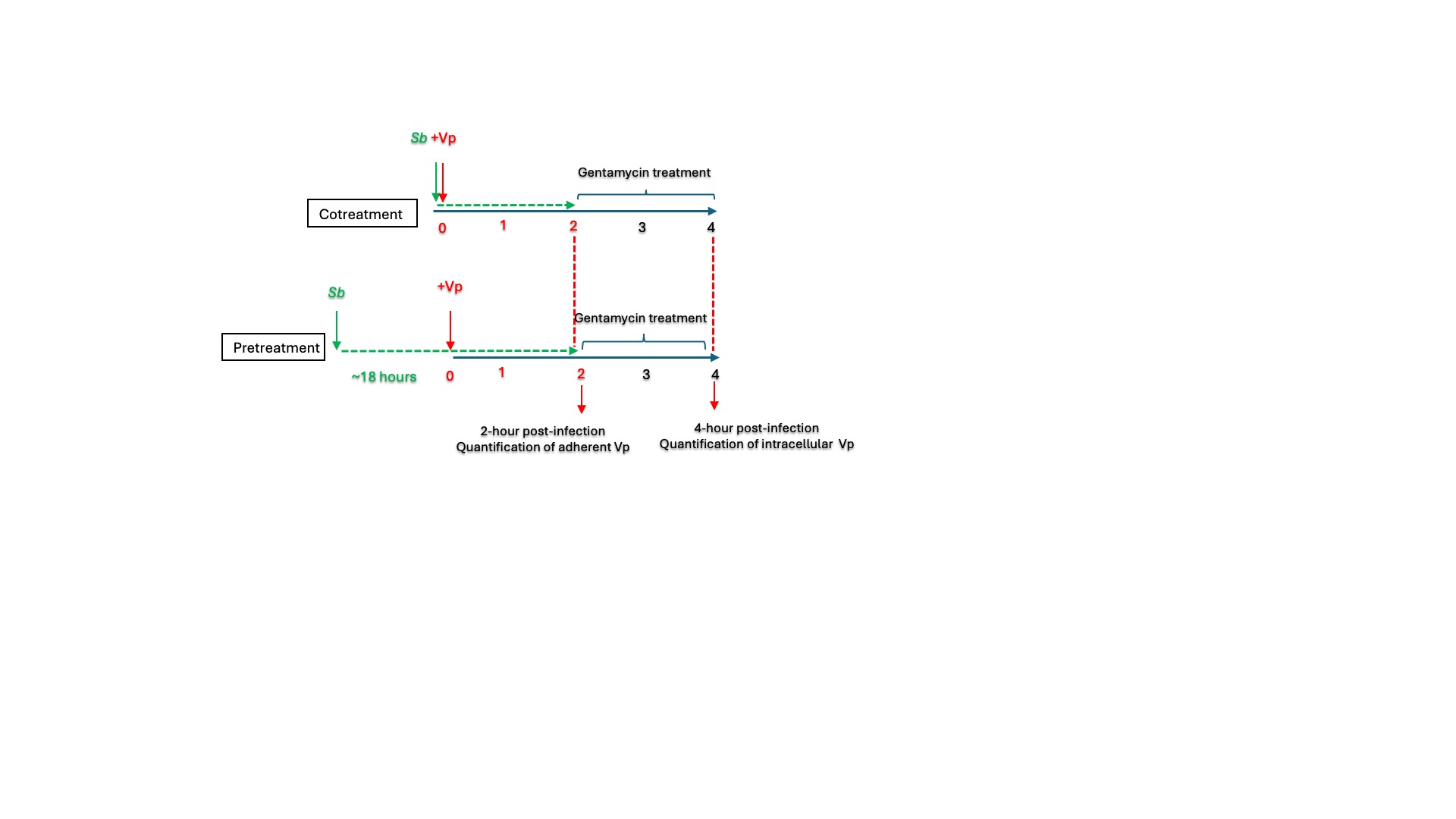

Supplement: Supplementary file 2 [file Image_2.jpeg]

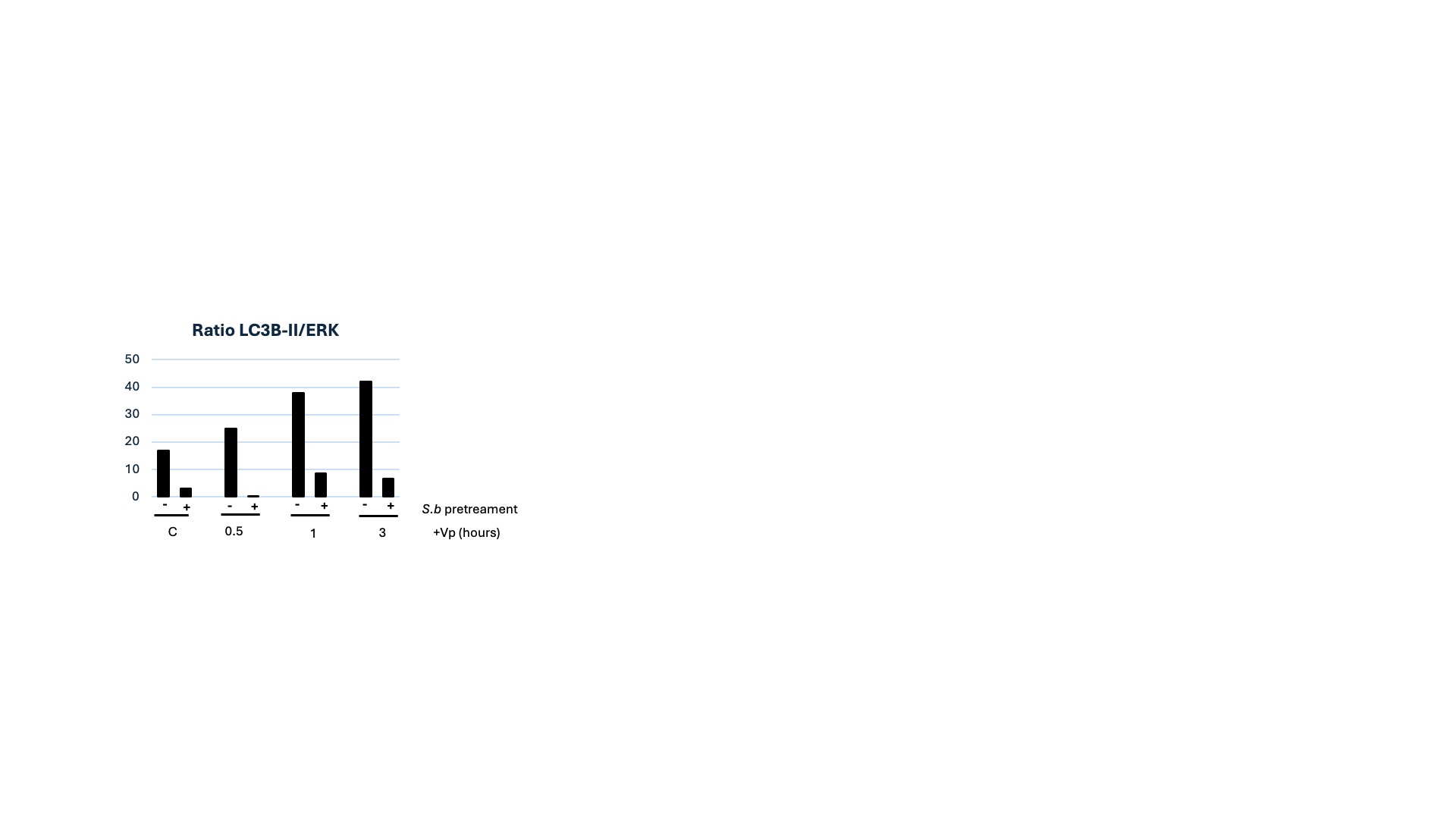

Supplement: Supplementary file 3 [file Image_3.jpeg]

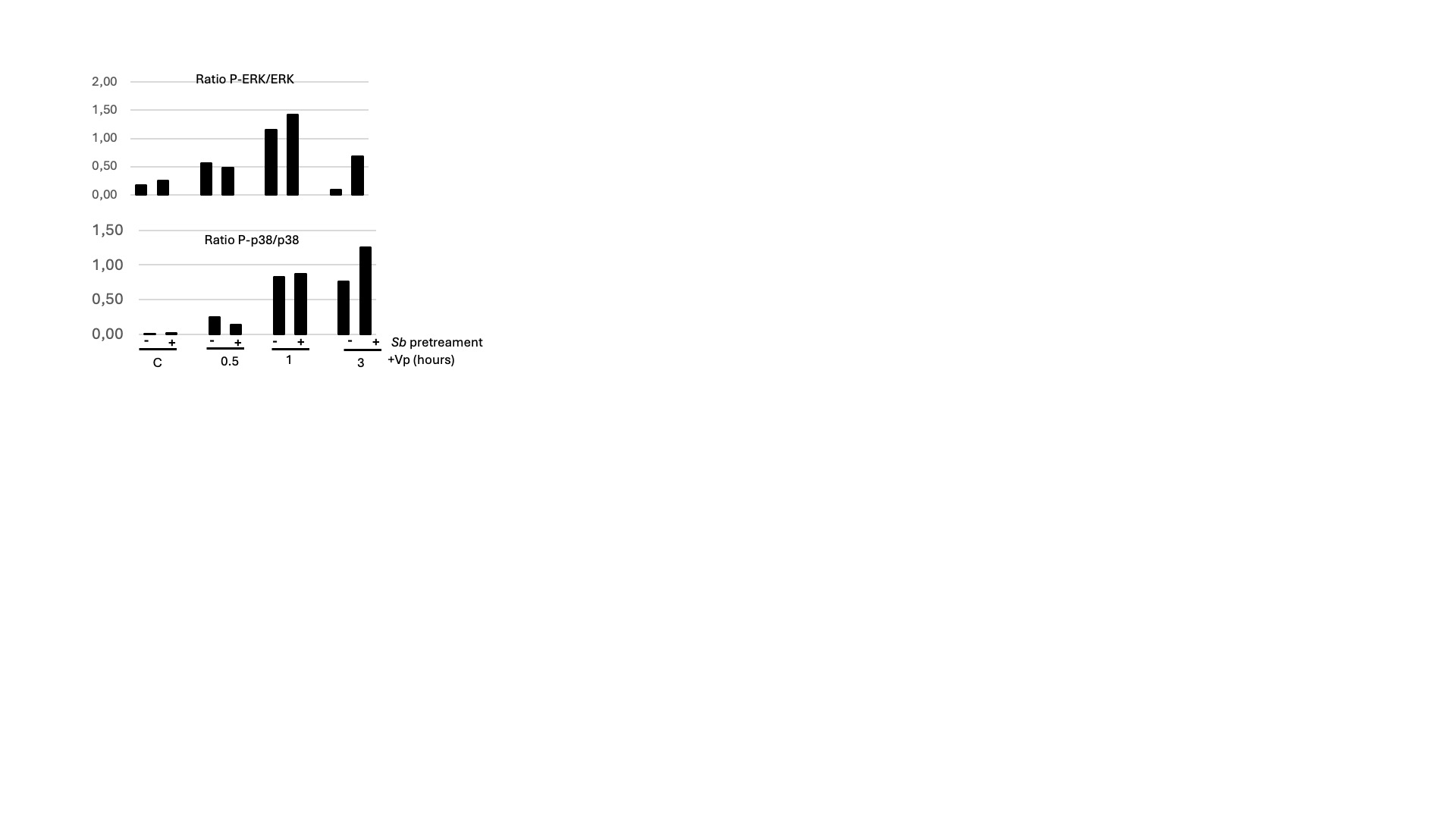

Supplement: Supplementary file 4 [file Image_4.jpeg]
